# Discoloration and Dilemma: A Case Report of Hand-Foot Syndrome Associated With Capecitabine Use

**DOI:** 10.7759/cureus.65151

**Published:** 2024-07-22

**Authors:** Rucha Gohil, Kalyan Bojanapati, Keerthi Reddy, Kavin Kumar, J Kumar

**Affiliations:** 1 General Medicine, SRM Medical College Hospital and Research Centre, SRM Institute of Science and Technology (SRMIST), Chengalpattu, IND

**Keywords:** hand-foot syndrome, chemotherapy, chemotherapy-related toxicity, palmoplantar dermatoses, carcinoma, rectosigmoid carcinoma, capecitabine, capecitabine-induced

## Abstract

This case highlights the occurrence of hand-foot syndrome due to the use of an antimetabolite group of drugs, capecitabine, which was used in the chemotherapy of a 56-year-old male patient who was diagnosed with rectosigmoid carcinoma. The patient was diagnosed with rectosigmoid carcinoma two months ago and underwent laparoscopic lower anterior resection and colorectal anastomosis. Subsequently, the patient commenced chemotherapy treatment with a combination of oxaliplatin and capecitabine. The patient presented to us with complaints of loose stools for the past three days, and discoloration of the palms, soles, and tongue was noted and subjected to a biopsy, which revealed features compatible with chronic, nonspecific dermatitis. The occurrence of such palmar-plantar erythrodysesthesia with capecitabine is yet to be extensively studied.

## Introduction

Capecitabine belongs to the antimetabolite group of drugs used as chemotherapeutic agents. It is a prodrug that is absorbed from the mucosa of the gastrointestinal tract, getting rapidly metabolized into a compound called 5-fluorouracil, which occurs in a three-step enzymatic process [1]. 5-Fluorouracil is responsible for cytotoxic activity by inhibiting the synthesis of DNA, inhibiting the processing of RNA, and thus inhibiting the synthesis of protein, resulting in cellular damage and adverse effects. Hand-foot syndrome is a condition characterized by erythema, edema, burning sensation, or discoloration of the palms and soles, sometimes associated with chemotherapy. Here, we report a case of hand-foot syndrome due to the use of an antimetabolite group of drugs.

## Case presentation

**Figure 1:**
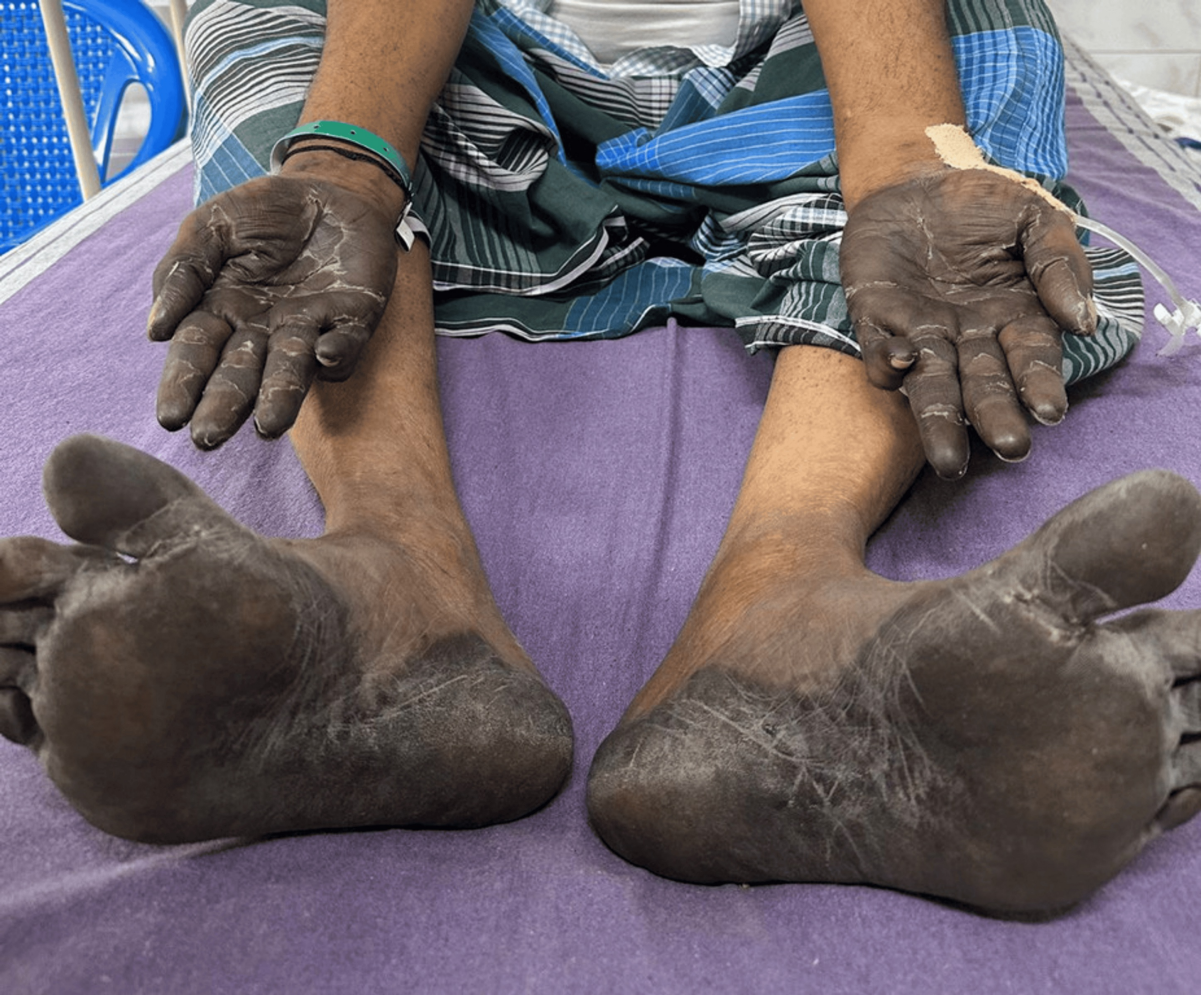
The palms and soles are exhibiting discoloration, indicating Grade 1 hand-foot syndrome.

A 56-year-old male patient who was diagnosed with carcinoma in the rectosigmoid colon two months ago on chemotherapy presented to us with the chief complaint of loose stools for the past three days. The patient was apparently well three days ago but subsequently experienced loose stools characterized by watery consistency, nonfoul smelling, not blood-tinged, and yellowish-green in color. He had 15-20 episodes per day. There was no history of associated vomiting, abdominal pain, or giddiness. There was discoloration noted in both the palms and soles by the patient for the past seven days (Figure 1).

**Figure 2:**
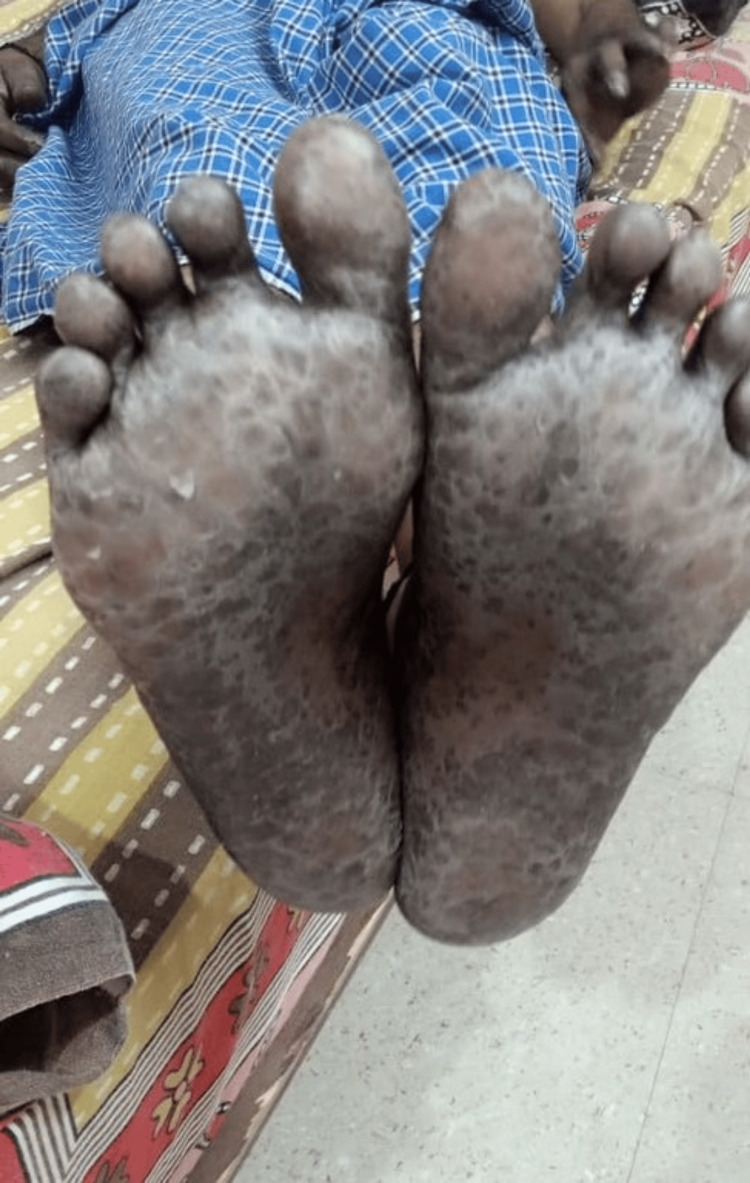
The feet show resolution of the skin lesions.

**Figure 3:**
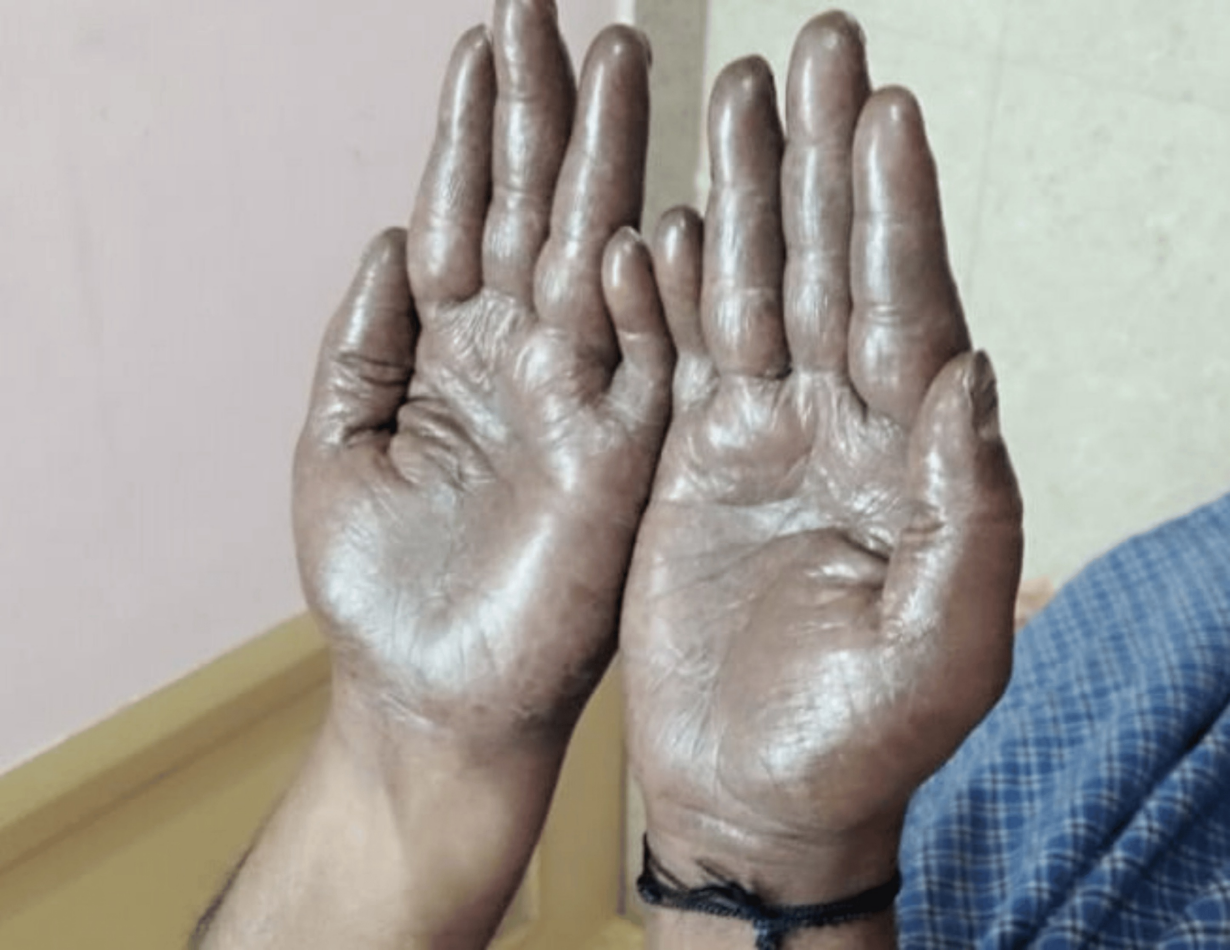
The palms exhibit a visible reduction in hyperpigmentation.

The patient was previously admitted under the care of the surgical gastroenterology department, where he underwent laparoscopic lower anterior resection and colorectal anastomosis and was then subjected to further treatment with chemotherapy. The patient received two cycles of intravenous administration of oxaliplatin at a dosage of 200 mg and was currently taking capecitabine tablets at 500 mg 3-0-3. The patient had no other co-morbidities. The patient was managed adequately with intravenous fluids and other symptomatic management, such as adequate hydration, replenishment of electrolytes, and zinc supplementation. The patient was subjected to a skin biopsy in view of hyperpigmentation noted on both the feet and palms, which revealed features compatible with chronic nonspecific dermatitis. The occurrence of palmar-plantar erythrodysesthesia and loose stool was found to be caused by the administration of capecitabine. As a result, the medication was withheld, and the patient received symptomatic treatment, including topical moisturizers. The patient's lesions showed signs of improvement (Figures 2, 3).

## Discussion

Hand-foot syndrome, which affects 3% to 64% of patients, is a frequent dermatologic reaction noted to chemotherapy, the highest rates of which are seen while using capecitabine (50%-60%) and doxorubicin (40%-50%) [2]. The mechanism of this syndrome remains unclear, one of which explains the occurrence of this syndrome because of the extravasation of these drugs through the tiny capillaries, mainly of the palms and feet, leading to a COX-induced inflammatory reaction or due to enzymes involved, such as thymidine phosphorylase and dihydropyrimidine dehydrogenase in the capecitabine metabolism [3].

The hands and feet are particularly prone to trauma from daily activities, resulting in tissue damage both locally and underneath the skin and due to unique attributes, including increased exposure to temperature changes, distinct microvascularization, abundant keratinocytes, eccrine glands, and a continual turnover of epidermal cells. Capecitabine was found to significantly induce the death of keratinocyte cells in vitro by activating the pathway of apoptosis and decreasing the potential of the mitochondrial membrane. These findings suggest that this syndrome due to capecitabine may result from the thinning of the corneous layer due to intracellular mitochondrial dysfunction and activation of the caspase-dependent apoptosis pathway [4]. Some propose that specific chemotherapeutic agents might enhance the activity of melanocyte-stimulating hormone, potentially contributing to the increased occurrence of this side effect in patients with darker skin tones [5].

The stages of hand-foot syndrome are divided into mild, moderate, and severe. Grade 1 includes minimal skin changes or dermatitis in the form of erythema, edema, or hyperkeratosis with no associated pain. Grade 2 includes skin changes with pain, limiting activities of daily living. Grade 3 includes severe skin changes consisting of peeling, blisters, fissures, bleeding, edema, or hyperkeratosis with pain, limiting self-care activities of daily living. The incidence of Grades 2 and 3 hand-foot syndrome was 26.8%. Independent predictive factors for developing these grades of this syndrome include age over 60 or more, a dosage of capecitabine that exceeds 3000 mg/day, and a patient who requires five or more cycles in the overall capecitabine treatment regimen. This information can be useful for clinical monitoring and helpful for the follow-up of patients [6].

In our current case scenario, the patient had blackish discoloration with irregular edges spread over the dorsum of the tongue and oral cavity, involving the buccal mucosa, inner lip regions, bilateral palms, and soles. The condition did not require any treatment as it did not hinder the patient's daily activities, except for causing some aesthetic concerns. It was a Grade 1 hand-foot syndrome. The improvement noted in the skin changes when the medication was discontinued was significant, which confirmed that hyperpigmentation of hand-foot syndrome was associated with the use of the drug capecitabine. Although our patient's symptoms improved after discontinuing capecitabine, it may not always be advisable to cease this medication in other cases, as it is still an effective treatment for colorectal cancer. Additionally, prophylaxis for hand-foot syndrome is an evolving option within oncology. Approaches can include the use of pyridoxine and formulations made of topical urea or topical lactic acid, although they are not as effective as celecoxib, which shows better potential in providing prophylactic efficacy. Ultimately, the selection of a treatment protocol should be made on an individual case basis, depending on the patient’s specific circumstances, prognostic predictors, and presentation [7].

Additional therapy and prophylactic strategies investigated include the D-TORCH research, which aims to evaluate the effectiveness of 1% topical diclofenac in lowering the occurrence of this illness [8]. Additionally, a thorough investigation was carried out in Japan that analyzed the impact of topical hydrocortisone butyrate 0.1% in conjunction with conventional moisturizing therapy to assess the frequency of hand-foot syndrome [9].

The optimal approach to managing hand-foot syndrome involves prolonging the interval between capecitabine administrations, reducing the dosage, or temporarily halting treatment until the toxicity diminishes to Grade 1 or lower. Additional strategies for addressing hand-foot syndrome encompass the application of topical moisturizers, antibiotics to ward off secondary infections, and topical corticosteroids, vitamin B6, and COX-2 inhibitors [10].

## Conclusions

Recognizing and comprehending the side effects of antineoplastic medications is crucial, given the importance of managing them to promote effective therapy and maintain the quality of the patient's life throughout and following antineoplastic therapy. Palmar-plantar and oral hyperpigmentation resulting from capecitabine usage and its correlation with hand-foot syndrome is an acknowledged phenomenon, yet further research is warranted in this area to deepen understanding, resolve uncertainties, and offer more insights into its pathophysiology. It is our aspiration that this case study adds to the expanding body of literature on refining hand-foot syndrome symptomatology and serves as a diagnostic aid for the prompt recognition and treatment of symptoms of hand-foot syndrome.
